# The Role of Progesterone and a Novel Progesterone Receptor, Progesterone Receptor Membrane Component 1, in the Inflammatory Response of Fetal Membranes to *Ureaplasma parvum* Infection

**DOI:** 10.1371/journal.pone.0168102

**Published:** 2016-12-15

**Authors:** Liping Feng, Carla E. Ransom, Matthew K. Nazzal, Terrence K. Allen, Yi-Ju Li, Tracy Truong, Lauren C. Potts, Patrick C. Seed, Amy P. Murtha

**Affiliations:** 1 Department of Obstetrics and Gynecology, Duke University, Durham, North Carolina, United States of America; 2 Department of Anesthesiology, Duke University, Durham, North Carolina, United States of America; 3 Department of Biostatistics and Bioinformatics, Duke University, Durham, North Carolina, United States of America; 4 Department of Pediatrics, Duke University, Durham, North Carolina, United States of America; Xavier Bichat Medical School, INSERM-CNRS - Université Paris Diderot, FRANCE

## Abstract

*Ureaplasma parvum* (*U*. *parvum*) is gaining recognition as an important pathogen for chorioamnionitis and preterm premature rupture of membranes. We aimed to investigate the roles of progesterone (P4) and a novel progesterone receptor, progesterone receptor membrane component 1 (PGRMC1), in the response of fetal membranes to *U*. *parvum*. Fetal membrane cells (amnion, chorion and decidua) were isolated and confirmed to be free of *Mycoplasmataceae*. Cells were treated with *U*. *parvum* (5x10^6^ CFU), and adherence was quantified by qPCR. Amnion and chorion cells were transfected with scrambled siRNA or validated PGRMC1 siRNA for 72h. Cells were then treated with *U*. *parvum* for 4h with or without pretreatment with P4 (10^−7^ M) or ethanol for 1h. Interleukin-8 (IL-8), matrix metalloproteinase 9 (MMP9) and cyclooxygenase (COX-2) mRNA expression were quantified by qRT-PCR. Culture medium was harvested and analyzed for IL-8 and prostaglandin (PGE_2_) secretion by ELISA and MMP9 activity by zymography. *U*. *parvum* had a mean adherence of 15.0±0.6%, 16.9± 3.7% and 4.7±0.3% in cultured amnion, chorion and decidua cells, respectively. Exposure to *U*. *parvum* elicited significant inflammatory responses including induction of IL-8, COX-2, PGE2 and MMP9. A possible role of PGRMC1 was identified in the inhibition of *U*. *parvum-*stimulated COX-2 and MMP9 mRNA expression in chorion cells and MMP9 activity in amnion cells. On the other hand, it might enhance the *U*. *parvum-*stimulated IL-8 protein secretion in amnion cells. P4, mediated through PGRMC1, significantly inhibited *U*. *Parvum*-induced MMP9 mRNA and COX-2 mRNA expression in chorion cells. P4 appeared to attenuate *U*. *parvum* induced IL-8 mRNA expression in chorion cells, but this P4 effect might not mediated through PGRMC1. In summary, *U*. *parvum* preferentially adheres to and induces inflammatory responses in chorion and amnion cells. P4 and PGRMC1 appear to differentially modulate the inflammatory responses induced by *U*. *parvum* among amnion and chorion cells.

## Introduction

Preterm birth (PTB) and preterm premature rupture of membranes (PPROM) remain major public health problems worldwide [1]. Although the exact mechanisms of PTB and PPROM are not well understood, infection of the fetal membranes has been implicated as an early event in their pathogenesis [2–4]. Bacteria are present in the fetal membranes irrespective of gestational age, labor, or rupture status. Specifically, bacteria are more abundant in fetal membranes collected from PPROM subjects compared to fetal membranes collected from term and preterm subjects [5].

*Ureaplasma* spp. are among the organisms most frequently implicated in prematurity-linked conditions and chorioamnionitis [6–11]. The two species of *Ureaplasma* known to colonize humans are *U*. *urealyticum* (serovars 2, 4, 5, 7–13) and *U*. *parvum* (serovars 1, 3, 6 and 14). Of these, *U*. *parvum* is the most common species isolated from the genital tract of men and women [6, 12]. Increasing evidence suggests that *U*. *parvum* is also an important pathogen in pregnancy and is associated with PPROM, PTB, and chorioamnionitis. *U*. *parvum* is the most frequently isolated pathogen in the amniotic fluid of women who deliver preterm [12], and *U*. *parvum* colonization in neonates is inversely related to gestational age at delivery [13]. The most recent study of the human microbiota during pregnancy indicated that elevated vaginal *Ureaplasma* abundances were associated with PTB [14]. The strongest evidence linking *U*. *parvum* to preterm labor is from experiments in animal models. Intra-amniotic inoculation of *U*. *parvum* resulted in chorioamnionitis in Rhesus macaques [15] and sheep [16] and also promoted preterm delivery in Rhesus macaques [15]. However, to date, the pathogenicity of *U*. *parvum* and host susceptibilities to *U*. *parvum* in fetal membranes are poorly understood [17].

In the Rhesus macaque model of *U*. *parvum* intrauterine infection, the onset of preterm labor was preceded by a rise in amniotic fluid leukocytes, inflammatory cytokines including interleukin-8 (IL-8), prostaglandins E_2_ and F_2α_ (PGE_2_ and PGF_2α_) and matrix metalloproteinase 9 (MMP9) [15]. These data were corroborated in a recent study using human fetal membrane explants in which IL-8 production and MMP9 activity were induced after exposure to *U*. *parvum* [18]. IL-8 has been implicated in the initiation of infection-mediated preterm labor [19]. Cyclooxygenase (COX-2) is important for the production of prostaglandins (PGs), including PGE_2_, which are synthesized in the fetal membranes and stimulates cervical softening and uterine contractions during labor [20]. Up-regulation of COX-2 is associated with labor and specifically may play a role in parturition complicated by intrauterine infection [21]. Additionally, MMPs cause remodeling of fetal membranes before the onset of labor and in PPROM. Both clinical and molecular studies have demonstrated the critical role of MMP9 in fetal membrane rupture [22–24]. Thus, we have chosen to evaluate IL-8, COX-2, and MMP9 as biomarkers of the inflammatory response of fetal membranes to *U*. *parvum* infection.

Progestins have long been used in the therapeutics of PTB. 17 alpha-hydroxyprogesterone caproate (17P) reduces the risk of recurrent PTB, and progesterone (P4) reduces the risk of PTB in the setting of short cervix [25, 26]. The immunomodulatory function of P4 has been studied primarily within the context of pregnancy and in the susceptibility to infections [27–29]. The influence of P4 on susceptibility to bacterial infection, including *U*. *parvum*, is unknown. In many tissues and cell types, the physiologic effects of progesterone are primarily mediated through nuclear progesterone receptors (PGRs). However, the two major isoforms of PGRs, progesterone receptor A and B, are not present in the amnion and chorion layers of fetal membranes, indicating potential PGR- independent mechanisms of progesterone [30]. A novel membrane progesterone receptor, progesterone receptor membrane component 1 (PGRMC1), is highly expressed and actively regulated in amnion and chorion cells [31]. PGRMC1 is a single transmembrane protein that has a high affinity for P4 with a low affinity for other steroids [32, 33]. Clinical and molecular studies from our group suggest potential roles of PGRMC1 in mediating progesterone function in fetal membranes and in maintaining fetal membrane integrity [31, 34].

Overall, our objective in this work was to expand the model of how *U*. *parvum* infection may invoke PTB and PPROM by inducing inflammation in the fetal membranes. Additionally, we sought to investigate the role of P4 and PGRMC1 in the inflammatory responses of fetal membrane cells to *U*. *parvum* infection. *U*. *parvum* adherence to host cells or tissues is a virulence factor and an essential step of *U*. *parvum* infection [35]. However, *U*. *parvum* adherence in gestational tissues including fetal membranes has not yet been closely studied. We hypothesized that *U*. *parvum* would have different tropism for cellular types of the fetal membranes, thus resulting in different host-pathogen interactions and inflammatory consequences among each cell type. We hypothesized that *U*. *parvum* exposure induces P4-sensitive IL-8, COX-2, PGE2, and MMP9 responses in the cells of the fetal membranes. And P4 acts through non-genomic progesterone receptor, PGRMC1. To test our hypothesis, we measured *U*. *parvum* adherence to the different cell types of the fetal membranes. We evaluated the inflammatory responses following *U*. *parvum* exposure as indicated by the biomarkers IL-8, COX-2, PGE2, and MMP9. We also investigated the role of P4 on this inflammatory response by pretreatment with P4 before *U*. *parvum* exposure and the role of PGRMC1 using a PGRMC1 knock-down primary cell model. These data describe a model for *U*. *parvum* interactions with the fetal membranes and the induction of an inflammatory cascade that may be a pathway to PPROM and PTB.

## Materials and Methods

This project was approved by the Duke University Institutional Review Board (IRB). Duke IRB approved the waiver of consent to obtain de-identified tissue that would not be used for clinical purposes.

### Bacterial strain and culture conditions

*U*. *parvum* strain 700970, SV3, a fully sequenced genital isolate, was obtained from the American Type Culture Collection. *U*. *parvum* was routinely cultured in 10B media [PPLO broth (BD, Sparks, MD), pH 6, supplemented with yeast extract (1% final), urea (0.1% final), l-cysteine (100 mg), ampicillin (100 micrograms/ml final), phenol red (10mg), isovitalex (0.25g, BD, Sparks, MD), and 20% equine serum (Equitech-Bio, Kerrville, TX)]. For consistency through all the experiments, aliquots of *U*. *parvum* from a single culture were frozen at -80°C and thawed as needed. For each experiment, a *U*. *parvum* aliquot was inoculated and grown in 10B media for 24 hours and pelleted by centrifugation (20,000 x g, 4°C) for 30 minutes. The *U*. *parvum* pellet was re-suspended in cell culture media for subsequent use.

### Fetal membrane collection and primary cell culture

Fetal membranes were collected following scheduled, uncomplicated cesarean delivery at term without rupture of membranes or labor. Institutional review board approval was obtained for waiver of consent to obtain de-identified tissue that would not be used for clinical purposes. Harvested tissue was transported to the laboratory at room temperature in Dulbecco's Modified Eagle Medium-Hams F12 (DMEM/F12) cell culture medium containing FBS (10%, v:v), penicillin (200 U/ml), streptomycin (200 μg/ml) and amphotericin B (0.5 μg/ml).

Fetal membrane tissues were cut into 2 × 2-inch squares with forceps and scalpel. The smooth layer of amnion was removed manually. Amnion cells were harvested using a modified technique which was previously described by Casey [36]. Briefly, the amnion tissue was minced into small fragments using 2 scalpel blades and was then digested in DMEM/F12 containing trypsin (Sigma Aldrich, St. Louis, MO) at 37°C for 30 minutes with periodic agitation. Thereafter, the mixture was filtered using a tissue strainer to separate the dispersed amnion epithelial cells from the tissue fragments. The epithelial cells were pelleted by centrifugation and re-suspended in DMEM/F12 medium. This process was repeated three times, and the dispersed epithelial cells were combined and counted. Viability was assessed by trypan blue dye exclusion (Invitrogen, Grand Island, NY), and the cells were plated in plastic culture plates in DMEM/F12 media with 10% FBS and antibiotic and anti-mycotic agents (Invitrogen, Grand Island, NY). The yield of amnion epithelial cells was 8–12 million/g of amnion tissue; viability was 90%. The cells replicated to confluence in about 7 days.

Separation of the decidua and chorion involved blunt dissection with forceps and scalpel. Chorion and decidua layers were minced by cross cutting with scalpel blades. Tissues were processed in digestion buffer (0.125% trypsin and 0.2% collagenase [Sigma Aldrich, St. Louis, MO]) at 37°C for about 90 minutes with periodic agitation. Cells were filtered through four layers of sterile gauze and centrifuged at 2000 rpm for 10 minutes. A cell-separation gradient was prepared with an Optiprep column (Sigma Aldrich, St. Louis, MO) to further purify chorion and decidua cells. Details of cell purification were described in our previous publication [37]. Cells were then plated in the same culture conditions as amnion cells for 48 hours.

Purity of primary amnion, chorion and, decidua cells were confirmed using immunofluorescence staining for cytokeratin (biomarker of amnion and chorion cells) and vimentin (biomarker of decidua cells). Cultures on glass chamber slides were fixed with cold methanol (-20°C) for 5–10 minutes. The cells were permeabilized and blocked with 1% BSA, 5% normal goat serum and 0.1% tween-20 in PBS for 60 minutes at room temperature. After blocking, the cells were incubated with primary antibodies overnight at 4°C in humidified chambers. Primary anti-cytokeratin and anti-vimentin mouse monoclonal antibodies (Dako, Carpinteria, CA) were used at 1:200. To demonstrate the expression of PGRMC1 in these cells, anti-PGRMC1 rabbit polyclonal antibody (Sigma Aldrich, St. Louis, MO) was used at 1:100 at the same time. Anti-mouse and rabbit IgG antibodies were used as negative control (R & D system, Minneapolis, MN). Goat anti-mouse secondary antibody Alexa Fluor 488 conjugate and goat anti-rabbit secondary antibody Alexa Fluo 594 (Life Technologies, Carlsbad, CA) were used at 1:500. Slides were mounted using mounting medium for fluorescence with DAPI (Vector Laboratories, Burlingame, CA) and examined with a Zeiss Axio Imager widefield fluorescence microscope.

Before treatments, all cell types were confirmed to be free of *Mycoplasma* and *Ureaplasma* contamination using a chemiluminescent-labelled single-stranded DNA probe hybridization method (MTC-NI kit, Millipore).

### Adherence assay of *U*. *parvum* to fetal membrane cells

To define the limitation in the assay conditions, treated cells were evaluated for cytotoxicity of *U*. *parvum* using a lactate dehydrogenase (LDH) release assay. Cytotoxicity was observed after 6 hours or longer exposure to *U*. *parvum* at the highest multiplicity of infection (MOI). MOI is the ratio of the number of bacteria (*U*. *parvum*) cells to the number of targeted cells (fetal membranes cells). On this basis, subsequent experiments were limited to 4 hours. Adherence assays were performed using a variation of the methods previously described by Smith et al [38]. Briefly, primary cells from the chorion, amnion and decidua were grown to 90% confluence, washed three times with warmed PBS + 0.01% Tween-80, and blocked with warmed PBS + 3% BSA for 60 minutes (37°C, 5% CO2). One ml of cell culture media (DMEM/F12 + 3% BSA + antibiotic and anti-mycotic agents) was then added for a further blocking step (60 minutes, 37°C, 5% CO2). *U*. *parvum* was cultured and prepared as described previously. *U*. *parvum* suspensions were added to cells at various MOI. After incubation (60 minutes, 37°C, 5% CO2), non-adherent *U*. *parvum* was removed by washing three times with warmed PBS + 0.01% Tween-80. Primary cells with adherent *U*. *parvum* were solubilized by the addition of 100 μl PBS containing 0.5% Nonidet P40 (15 minutes, 20°C, shaker) and were lifted by scraping. The lysis solution was collected and analyzed for genome copy number of *U*. *parvum* by quantitative PCR (qPCR). All assays were carried out in triplicate. Adherence is expressed as the percentage of *U*. *parvum* remaining bound to the tissue culture cells from the total inoculum.

### Real-time PCR for quantification of *U*. *parvum*

#### Isolation of genomic DNA of *U*. *parvum*

*U*. *parvum* pellets were re-suspended in nuclei lysis buffer (20 mM tris at pH 8.0, 10 mM EDTA, and 50 mM NaCl) with 1% sodium dodecyl sulfate (SDS). Proteinase K was added to make a final concentration of 120 μg/ml solution, and the pellets were incubated at 60°C for 45 minutes. After 10 minutes at room temperature, 6M NaCl was added to precipitate the protein. Centrifugation (~12000 x g) was performed at 4°C for 10 minutes until a pellet was seen. The supernatant was transferred to a new tube, and equal volume isopropanol was added until DNA threads were visible. DNA was collected by centrifugation (~12000 x g) for 10 minutes at 4°C, and the supernatant was aspirated. The nucleic acid pellet was washed with 70% ethanol and centrifuged. The ethanol was aspirated, and the pellet was re-suspended in Tris-EDTA (TE) buffer (10 mM Tris pH 8.0 and 1 mM EDTA) with RNase and maintained at 4°C overnight. Next, a sample of the nucleic acid suspension was analyzed via NanoDrop Spectrophotometer (NanoDrop, Wilmington, DE). Agarose gel electrophoresis was used to confirm the purity of *U*. *parvum* DNA samples [39].

#### Quantitative PCR

For the determination of *U*. *parvum* cell numbers, a quantitative dye intercalation PCR assay was designed to measure the conserved, single genomic copy of *U*. *parvum* urease subunit *ureB* gene (Ensembl Genome Browser). Primers were as follows: ureB-RT-L.1 (GGACGACGTTTCGATATTCC) and ureB-RT-R.1 (ACCTCAAACTTCGCGTGTTC). *UreB* gene sequence, amplification region and amplicon size are listed in a supplement file. Real-time PCR assays were performed on an iCycler with an optical module (Bio-Rad, Hercules, CA). Components of the PCR master mix included: 2.5 μl of 10x buffer, 1.25 μl of 50 μM MgCl_2_ (2.5 μM), 0.3μl of dNTP mix (200 μM), 0.5 μl Taq Apex^™^ DNA polymerase (Genesee Scientific, RTP, NC), 0.2 μl each of forward and reverse primer (100 pg), 1.25 μl Evagreen dye (20x; Biotium, Candler, NC), and 16.8 μl purified water. A volume of 2 μl of the specimen was added to 23 μl of the master mix for a total volume of 25 μl. The iCycler was set to the following program: 10 minutes pre-incubation at 95°C; 40 cycles of amplification (15 seconds at 95°C, 30 seconds at 55°C, 15 seconds at 72°C, and 15 seconds at 82°C with a single fluorescence measurement); 15 seconds at 95°C; and 30 seconds at 55°C. Melting curves were generated in continuous acquisition mode during interval changes of 0.1°C each 5 seconds from 70°C to 90°C. Finally the machine was cooled to 4°C. All PCR runs included a set of known copy standards, two experimental controls (*U*. *parvum* without tissue culture cells; tissue culture cells without *U*. *parvum*), and a PCR positive control of *U*. *parvum* in known genomic copies. Unknown sample copy numbers were derived by comparison against the standard curve. Size and purity of PCR products were verified on a 2% agarose gel.

### PGRMC1 siRNA transfection and experimental treatments

Primary cultured amnion and chorion cells were plated in 6-well plates and grown to 40–50% confluence. Cells were then transfected with scrambled siRNA (Ambion^®^, Life Technologies, Grand Island, NY, catalog no. AM4611) or predesigned PGRMC1 siRNA (Ambion^®^, Life Technologies, Grand Island, catalog no S21310) using Lipofectamine RNAiMAX (Invitrogen, Grand Island, NY) to yield a final concentration of 10 nM. The siRNA transfection was performed as outlined by the manufacturer. Twenty-four hours after transfection, cell lysates were harvested and processed for qPCR to determine knockdown of PGRMC1 mRNA expression. PCR condition was described in our previous publication [40]. Seventy-two hours after transfection, cell lysates were harvested and processed for Western blotting to determine knockdown of PGRMC1 protein expression. Housekeeping gene and protein glyceraldehyde 3-phosphate dehydrogenase (GAPDH) was used as an internal control for qPCR and Western blotting respectively. The primary antibody used for Western blotting was rabbit anti-human PGRMC1 antibody (1:2000, Sigma, St. Louis, MO) and rabbit anti-human GAPDH antibody (1:20000, Cell Signaling Technology, Beverly, MA). In parallel, after 72 hours transfection, the cells were treated with *U*. *parvum* (5x10^6^ CFU) with and without 1 hour pretreatment of P4 (10^−7^ M) or ethanol (vehicle). After 4 hours *U*. *parvum* exposure, conditioned cell culture medium was harvested for ELISA and gelatin zymography. Total RNA was extracted from the cells using RNeasy Mini Kit (Qiagen, Hilden, Germany).

### Real-time quantitative reverse transcription q(RT)-PCR

Total RNA concentration was quantified using NanoDrop Spectrophotometer (NanoDrop, Wilmington, DE). One ug of total RNA was used to generate double-stranded cDNA using SuperScript III and Oligo dT (Invitrogen, Grand Island, NY) following the manufacture’s protocols. Fifty ng of cDNA were used for real-time quantitative PCR using pre-validated Taqman^®^ gene expression probes targeted against MMP9 (Ambion^®^, Life Technologies, Grand Island, assay ID: Hs00234579_m1) and COX-2 (Ambion^®^, Life Technologies, Grand Island, assay ID: Hs00153133_m1). 2 x IQ supermix cocktail (Bio-Rad, Hercules, CA) was used for these probe assays. IL-8 and the housekeeping gene beta-2-microglobulin (B2M) mRNA expression level were measured by SYBR^®^ green detection method (Bio-Rad, Hercules, CA). 2 x IQ SYBR^®^ Green supermix cocktail (Bio-Rad, Hercules, CA) was used for these assays. Primers used for IL-8 are forward primer (ACTGAGAGTGATTGAGAGTGGAC) and reverse primer (AACCCTCTGCACCCAGTTTTC). Primers used for B2M are forward primer (TGCTGTCTCCATGT TTGATGTATCT) and reverse primer (TCTCTGCTCCCCACC TCTAAGT). The iCycler with optical module was programmed for an initial denaturation step of 95°C for 2 minutes, followed by a two-step amplification phase of 35 cycles of 95°C for 30 seconds, and 60°C for 1 minute while sampling for FAM emission. Data were analyzed using the iCycler platform software. Samples were run in duplicate, and the mean cycle thresholds (Ct) were normalized to the average B2M Ct. Fold changes were calculated using ΔΔCt method after normalization.

### Measurement of IL-8 and PGE_2_ protein concentrations by ELISA

Harvested cell culture media were immediately frozen at -80°C until analysis. The media were analyzed for IL-8 protein concentrations by Quantikine^®^ ELISA Kit and PGE_2_ protein concentrations by Parameter Assay Kit (R&D Systems, Minneapolis, MN) following manufacturer’s instructions. Samples without dilution were run with serial dilutions of recombinant human IL-8 and PGE_2_ as standards. The lower limit of sensitivity of ELISA for IL-8 was 3.5 pg/ml and PGE_2_ was 30 pg/ml. Samples were run in duplicate, and the absorbance was measured at optical density (OD) of 450 nm with correction at OD of 540 nm.

### Gelatin zymography for MMP9 activity

Gelatin zymography was used to quantify MMP9 activity *in vitro* using a protocol previously described [34]. All gels and reagents were purchased from Invitrogen (Grand Island, NY). Briefly, harvested cell culture medium was incubated in a 1:1 ratio with a Novex^®^ tris glycine sample reducing buffer and SDS sample loading buffer for 10 minutes at room temperature. Samples were loaded onto a 10% Novex^®^ gelatin zymogram gel and electrophoresed at 125 volts for 90 minutes. The gels were incubated in Novex^®^ renaturing buffer for 30 minutes and then Novex^®^ developing buffer for 30 minutes to allow the enzymes to re-nature. After incubation with fresh developing buffer for an additional 16 to 24 hours, the gels were washed with deionized water and stained with Simplyblue Safestain for 1 hour. The gels were de-stained by washing with deionized water for 2 hours at room temperature. MMP9 activity was quantified by analyzing band densities at 88 and 92 kDa using Image J^®^ (NIH) densitometry software.

### Data analysis

Primary cells harvested from one subject were treated either with scrambled siRNA or control siRNA (CsiRNA) or predesigned PGRMC1 siRNA (PsiRNA). Then treatments including *U*. *parvum* (UPA) alone, UPA with ethanol pretreatment (E+UPA), and UPA with P4 pretreatment (P4+UPA) were compared within and between groups. We performed all above experiments using primary cells from one subject. Sample sizes were counted as n subjects and presented in each figure as “n = x”. Data are presented as mean values ± standard errors of mean (SEM). Since cell groups (CsiRNA and PsiRNA) are derived from the same subject in each experiment run, we chose Generalized Estimating Equations (GEE) model for the analysis in order to take into account within subject correlation. GEE models with compound symmetry covariance structure were used to model cell groups, treatments (control [N], UPA, P4+UPA, E+UPA), and their interactions on outcomes. The pre-specified comparisons of interest were UPA versus control (N) and E+UPA versus P4+UPA within cell groups and across cell groups. These comparisons were conducted using contrast statements within PROC GENMOD in SAS. *P* < 0.05 was considered significant. All analyses were conducted using SAS 9.4 software (SAS Institute, Inc., Cary, NC).

## Results

**Fig 1:**
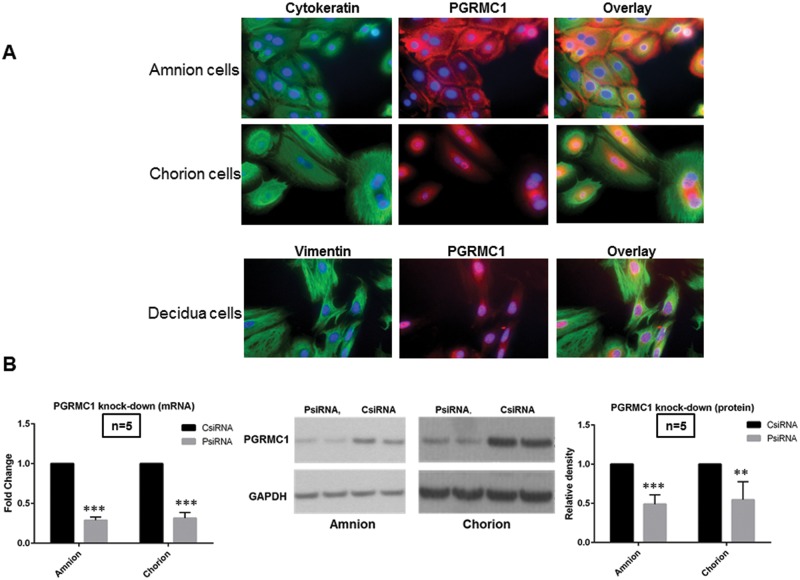
**A, Immunofluorescence staining of the biomarkers of fetal membrane cells and PGRMC1.** Above 95% of amnion and chorion cells stained positive for their biomarker cytokeratin (green). Amnion and chorion cells appear to be epithelial cell phenotype. Above 95% of decidua cells stained positive for their biomarker vimentin (green). Decidua cells demonstrated typical stromal cell phenotype. All cells stained positive for PGRMC1 (red). PGRMC1 protein in amnion cells is mainly localized to the cell membrane and cytoplasm. In contrast, the expression of PGRMC1 protein in chorion and decidua cells is mainly localized to nuclear and perinuclear space. DAPI counterstaining was performed to visualize nuclei (blue). Images of cells were captured using digital camera interfaced with a fluorescence microscope. Magnification is 40. **B, PGRMC1 mRNA and protein knock-down in amnion (A) and chorion cells (C).** PsiRNA is for PGRMC1 siRNA transfected group; CsiRNA is for control siRNA transfected group. qPCR results consistently showed about 70% knockdown of PGRMC1 mRNA production (PsiRNA vs CsiRNA, *P*<0.0001 for amnion cells, *P*<0.0001 for chorion cells, n = 5). Representative Western blotting and densitometry data are presented. Western blotting consistently showed above 50% knockdown of PGRMC1 protein production (PsiRNA vs CsiRNA, *P* = 0.0007 for amnion cells, *P* = 0.01 for chorion cells, n = 5).

Primary amnion, chorion and decidua cells were harvested and verified by immunofluorescence staining with cytokeratin (epithelial cell marker) and vimentin (stroma cell marker) (Fig 1A). As expected, PGRMC1 was expressed throughout each cell type [31, 34] but with different localization patterns. PGRMC1 protein in amnion cells was primarily localized to the cell membrane and cytoplasm. In contrast, the expression of PGRMC1 protein in chorion and decidua cells was found in the nuclear and perinuclear space. The depletion of PGRMC1 mRNA and protein by siRNA transfection from amnion and chorion cells was confirmed by qPCR and Western blotting respectively (Fig 1B).

### Adherence of *U*. *parvum* to primary amnion, chorion and decidua cells of the fetal membranes

**Fig 2:**
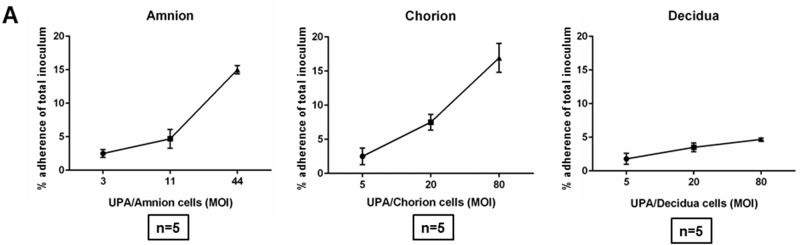
Adherence of *Ureaplasma parvum* (*UPA*) serotype 3 to fetal membrane cells (amnion, chorion and decidua). The percentage of adherence of *UPA* to each cell type increased as the MOI increased. At the maximal MOI, *UPA* had a significant higher adherence in primary amnion and chorion cells compared with decidua cells. (n = 5).

*U*. *parvum* was incubated with each cell type for 1 hour over a range of MOI. After extensive washing to remove unbound *U*. *parvum*, the number of adherent organisms was measured by qPCR against a standard curve for the single copy *U*. *parvum ureB* gene. *U*. *parvum* adherence to each cell type increased as the MOI increased (Fig 2). At the maximal MOI, *U*. *parvum* had a mean adherence of 15.0±0.6% in cultured primary amnion cells and 16.9± 3.7% in cultured primary chorion cells. In contrast, cultured decidua primary cells demonstrated an adherence of only 4.7±0.3% (Fig 2).

Together, the adherence data suggest a higher tropism by *U*. *parvum* for fetally-derived amnion and chorion cells. These data provided a rationale for using amnion and chorion cells in subsequent experiments.

### The effects of P4 pretreatment and PGRMC1 knockdown on IL-8 expression induced by *U*. *parvum* infection in amnion and chorion cells

To address the role of PGRMC1 in the inflammatory response to *U*. *parvum* and in P4 effects, PGRMC1 was depleted in primary amnion and chorion cells using siRNA transfection. Cells were treated with either PGRMC1-targeting siRNA (PsiRNA) or a scrambled control siRNA (CsiRNA). The control or PGRMC1-specifically targeted cells were then treated with either pretreated with vehicle control (ethanol) followed by *U*. *parvum* exposure (E+UPA) or pretreated with P4 followed by *U*. *parvum* exposure (P4+UPA). For mRNA results, UPA, (P4+UPA), and (E+UPA) groups were normalized to no treatment control (N) within CsiRNA and PsiRNA respectively. For protein results, concentrations were presented without normalization to any treatment groups.

**Fig 3:**
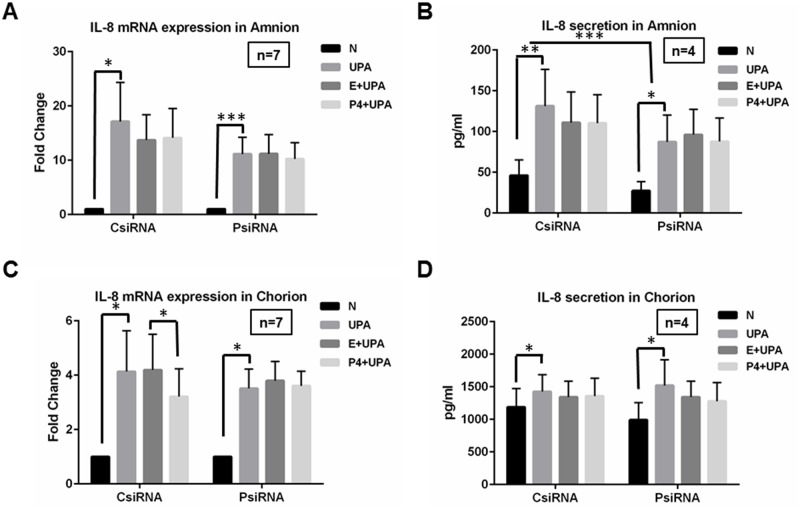
The effects of P4 pretreatment or PGRMC1 knockdown on IL-8 mRNA and IL-8 protein secretion induced by *U*. *parvum* infection in amnion and chorion cells. **A, B, C, D,** IL-8 mRNA expression and protein secretion were significantly up-regulated following *U*.*parvum* stimulation (UPA) compared to no *U*. *parvum* treatment control (N) in amnion and chorion cells (CsiRNA). **A,** No significant differences were observed for *U*. *parvum* induced IL-8 mRNA expression between CsiRNA and PsiRNA in amnion cells. **B,** but IL-8 protein secretion induced by *U*. *parvum* exposure appears decreased in PsiRNA. **C, D,** No significant differences were observed for *U*. *parvum* induced IL-8 mRNA expression and protein secretion when PGRMC1 was knocked down (PsiRNA) in comparison to CsiRNA in chorion cells. **A, B, C, D,** P4 pretreatment followed by *U*. *parvum* treatments (P4+UPA) partially inhibited the *U*. *parvum* induced up-regulation of IL-8 mRNA expression in chorion cells and had no effects on IL-8 mRNA and protein secretion compared to vehicle control group (E+UPA) in amnion cells. This P4 effect was not changed in PsiRNA cells compared with CsiRNA cells.

As shown in Fig 3A, IL-8 transcript levels were significantly induced by *U*. *parvum* exposure (UPA) in amnion cells compared to levels in unexposed cells (N) in CsiRNA group (*P* = 0.02), and PsiRNA treatment had no effect on IL-8 transcript levels following *U*. *parvum* exposure (*P* = 0.0004) compared to the CsiRNA treatment control cells (*P* = 0.16). P4 did not affect the *U*. *parvum* induced up-regulation of IL-8 mRNA expression compared to E+UPA in the amnion cells (CsiRNA: *P* = 0.79; PsiRNA: *P* = 0.16). The differences of E+UPA and P4+UPA between CsiRNA and PsiRNA were also not statistically significant (P = 0.39). IL-8 protein secretion was also significantly induced by *U*. *parvum* treatment in CsiRNA (*P* = 0.0005) and PsiRNA (*P* = 0.0064) groups (Fig 3B). The up-regulation of IL-8 protein secretion by *U*. *parvum* exposure was significantly different between the PsiRNA and CsiRNA groups (*P* = 0.0001). These data indicated that PGRMC1 enhanced *U*. *parvum* infection-induced IL-8 protein secretion in amnion cells. No significant differences were observed for any comparisons involved in P4 treatments (*P*>0.5, Fig 3B).

In chorion cells, significant up-regulation of IL-8 mRNA occurred following *U*. *parvum* exposure (UPA vs N) in both CsiRNA (*P* = 0.025) and PsiRNA (*P* = 0.0057) groups (Fig 3C). No significant differences were observed for *U*. *parvum* induced-IL-8 mRNA expression between CsiRNA and PsiRNA groups (*P* = 0.7). P4 pretreatment (P4+UPA) minimally attenuated the *U*. *parvum* induction of IL-8 mRNA expression compared to E+UPA in CsiRNA group (*P* = 0.026, Fig 3C). In PsiRNA group, the differences between E+UPA and P4+UPA were no longer observed (*P* = 0.64, Fig 3C). However, in comparison of the CsiRNA and PsiRNA groups, the differences of E+UPA and P4+UPA were not statistically significant (*P* = 0.12). These data demonstrated that P4 minimally inhibited IL-8 mRNA expression induced by *U*. *parvum*. However, if this P4 effect was mediated through PGRMC1 needs further investigation. IL-8 protein secretion was also stimulated by *U*. *parvum* treatment in both CsiRNA group (*P* = 0.008, Fig 3D) and PsiRNA (*P* = 0.029, Fig 3D). However, the depletion of PGRMC1 or P4 pretreatment did not significantly affect *U*. *parvum* induced IL-8 protein secretion in chorion cells (*P*>0.16, Fig 3D).

Ethanol or P4 alone did not affect IL-8 mRNA or protein secretion in these cells.

### The effects of P4 pretreatment or PGRMC1 knockdown on COX-2 mRNA expression and PGE_2_ secretion induced by *U*. *parvum* infection in amnion and chorion cells

**Fig 4:**
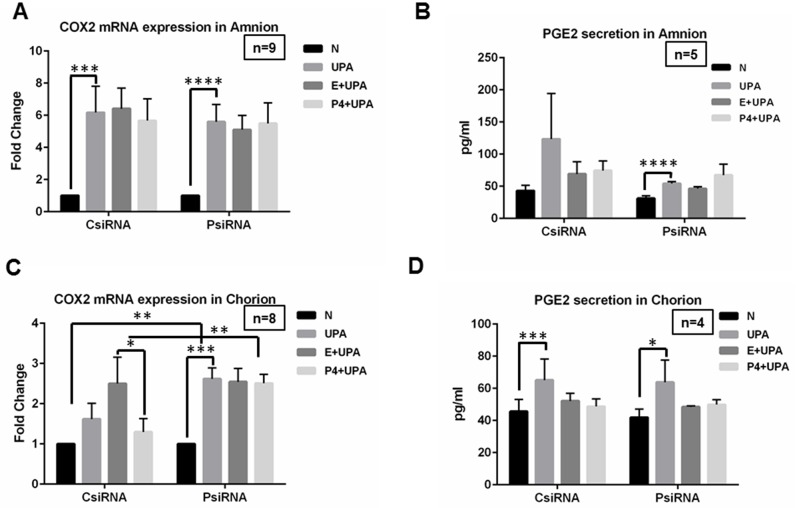
The effects of P4 pretreatment or PGRMC1 knockdown on COX-2 mRNA expression and PGE_2_ protein secretion induced by *U*. *parvum* infection in amnion and chorion cells. In amnion cells, **A, B,** significant up-regulation of COX-2 mRNA expression and PGE2 secretion induced by *U*. *parvum* was observed (N vs UPA). No significant differences of this up-regulation were observed between PsiRNA and CsiRNA groups. **A, B,** P4 pretreatment did not affect the *U*. *parvum* induced up-regulation of COX-2 mRNA level and PGE2 secretion compared to ethanol pretreatment (E+UPA vs P4+UPA). P4 effect was not different between PsiRNA and CsiRNA groups. **D**, similar trend was also observed for PGE2 protein secretion in chorion cells. **C,** the up-regulation of COX-2 mRNA expression by *U*. *parvum* exposure was significantly enhanced in PsiRNA cells in chorion cells. P4 pretreatment partially attenuated the *U*. *parvum* induced up-regulation of COX-2 mRNA expression compared to ethanol pretreatment (CsiRNA). The differences between E+UPA and P4+UPA disappeared in PsiRNA cells. The differences in the P4 effects between the CsiRNA and PsiRNA groups are statistically significant.

As shown in Fig 4, in amnion cells, *U*. *parvum* significantly induced COX-2 mRNA expression in both the CsiRNA (*P* = 0.0008) and PsiRNA (*P*<0.0001) groups (Fig 4A). No significant difference of this up-regulation (UPA vs N) was observed between PsiRNA and CsiRNA groups (*P* = 0.55, Fig 4A). No significant differences were observed for any comparisons involved in P4 treatments (*P*>0.19, Fig 4B).

Similar trends were observed for PGE2 protein secretion in amnion cells. PGE2 protein secretion was increased by *U*. *parvum* treatment in PsiRNA (*P*<0.0001) group, but the increase was not statistically significant in CsiRNA (*P* = 0.15) (Fig 4B). However, no significant differences were observed for any other comparisons described above (*P*>0.11).

In chorion cells, the up-regulation of COX-2 by *U*. *parvum* exposure was not significant in the CsiRNA (*P* = 0.08, Fig 4C). However, this effect was significantly enhanced in the PsiRNA (*P*<0.0001, Fig 4C). The up-regulation of COX-2 by *U*. *parvum* exposure was significantly different between the PsiRNA and CsiRNA groups (*P* = 0.0002, Fig 4C). These results demonstrate that PGRMC1 inhibits the COX-2 response to *U*. *parvum* exposure in chorion cells. P4 pretreatment (P4+UPA) partially attenuated the *U*. *parvum* induced up-regulation of COX-2 mRNA expression compared to E+UPA in the chorion cells in CsiRNA group (*P* = 0.03, Fig 4C). In PsiRNA, the difference between E+UPA and P4+UPA was no long observed (*P* = 0.65, Fig 4C). These differences of E+UPA and P4+UPA between CsiRNA and PsiRNA groups were observed (*P* = 0.01). These data demonstrate that P4 partially inhibited COX-2 mRNA expression by *U*. *parvum* and this P4 effect is mediated through PGRMC1.

PGE_2_ protein secretion was increased by *U*. *parvum* treatment in CsiRNA group (*P* = 0.0002) and PsiRNA group (*P* = 0.0047) in chorion cells (Fig 4D). However, the increased PGE_2_ protein concentration was not significantly influenced by P4 pretreatment or PGRMC1 knockdown (*P*>0.25).

### The effects of P4 pretreatment or PGRMC1 knockdown on increased MMP9 mRNA level and activity induced by *U*. *parvum* infection in amnion and chorion cells

For both MMP9 mRNA expression and MMP9 activity, *U*. *parvum* alone (UPA), UPA with P4 pretreatment (P4+UPA) and vehicle (Ethanol) control (E+UPA) groups were normalized to treatment with no treatment control (N) within each group (CsiRNA or PsiRNA).

**Fig 5:**
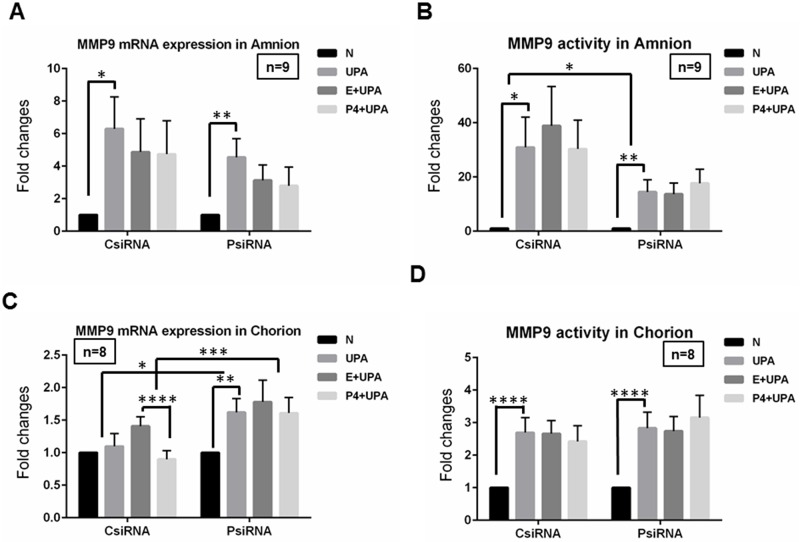
The effects of P4 pretreatment or PGRMC1 knockdown on MMP9 mRNA level and MMP9 activity induced by *U*. *parvum* infection in amnion and chorion cells. In amnion cells, **A and B,** *U*. *parvum* exposure significantly increased MMP9 mRNA expression and activity. The induction of MMP9 activity but not MMP9 mRNA was affected by PGRMC1 knock-down. The induction of MMP9 mRNA and activity by *U*. *parvum* exposure was not influenced by P4 pretreatment. In chorion cells, **C,** MMP9 mRNA expression was not significantly induced by *U*. *parvum* exposure in CsiRNA cells while it was significantly induced in PsiRNA cells. P4 pretreatment appeared to partially inhibit induced MMP9 mRNA expression by *U*. *parvum* exposure in CsiRNA cells. This effect of P4 was disappeared in PsiRNA cells. The differences of UPA vs N or (E+UPA) vs (P4+UPA) between CsiRNA and PsiRNA were statistically significant. **D,** MMP9 activity was significantly induced by *U*. *parvum* exposure in both CsiRNA and PsiRNA chorion cells, and this induction was not affected by either PGRMC1 knockdown or P4 pretreatment.

In amnion cells, *U*. *parvum* significantly induced MMP9 mRNA expression in both CsiRNA (*P* = 0.0043) and PsiRNA (*P* = 0.0001) groups (Fig 5A). No significant difference of this up-regulation was observed between PsiRNA and CsiRNA groups (*P* = 0.017, Fig 5A). No significant differences were observed for any comparisons involved in P4 treatments (*P*>0.16, Fig 5A). MMP9 activity was also increased by *U*. *parvum* treatment in CsiRNA (*P* = 0.0045) and PsiRNA (*P* = 0.0013) groups (Fig 5B). And significant difference of this induction was observed between PsiRNA and CsiRNA groups (*P* = 0.0499, Fig 5B). No significant effect was observed when comparing E+UPA and P4+UPA within CsiRNA or PsiRNA, and between CsiRNA and PsiRNA (*P*>0.23, Fig 5B)

In chorion cells, *U*. *parvum* significantly induced MMP9 mRNA in PsiRNA (*P* = 0.0008) group (Fig 5C). However, MMP9 mRNA expression was not changed in CsiRNA group (*P* = 0.38). Thus, these differences of UPA and N between CsiRNA and PsiRNA groups were observed (*P* = 0.05). These data indicated that knock-down of PGRMC1 in chorion cells may potentially enhance the induction of MMP9 mRNA by *U*. *parvum* exposure in chorion cells. In other words, PGRMC1 might protect chorion cells from induction of MMP9 mRNA by *U*. *parvum* exposure. P4 pretreatment significantly attenuated *U*. *parvum* exposure induced MMP9 mRNA expression in CsiRNA group (*P*<0.0001, Fig 5C). This P4 effect disappeared after PGRMC1 was knocked-down (*P* = 0.40, PsiRNA). These differences of E+UPA and P4+UPA between CsiRNA and PsiRNA groups were statistically significant (*P* = 0.0042). These data demonstrated that P4 inhibited MMP9 mRNA expression induced by *U*. *parvum* and the P4 effect is partially mediated through PGRMC1. *U*. *parvum* exposure also significantly stimulated MMP9 activity in chorion cells (*P*<0.0001, Fig 5D). No significant difference of any other comparisons regarding MMP9 activity in chorion cells was observed (*P*>0.12, Fig 5D).

## Discussion

*U*. *parvum* has been closely linked with preterm birth, leading to a hypothesis that it initiates and perpetuates an inflammatory cascade that ultimately leads to the induction of labor and membrane weakening. In this study, we probed this hypothesis using a primary cell culture model of human fetal membrane cells to study the *U*. *parvum–*host interaction and the inflammatory consequences.

As a first step in infection, *U*. *parvum* must localize and adhere to target host cells [41–43]. Previous reports describe *Ureaplasma* adherence to a variety of human cells including human erythrocytes (4–12%) [44] and human epithelial cells (1–20%) [38]. *U*. *parvum* surface-associated proteinaceous adhesins may be involved in the cytoadherence process, which has not yet been characterized in its entirety [38, 44, 45]. Our data indicate that *U*. *parvum* from genital isolated clinical strains adheres to human fetal membrane cells. Using assay that measures adherence of *U*. *parvum* by quantitative PCR, we demonstrated that *U*. *parvum* preferentially adheres to primary cultured amnion and chorion cells compared to decidua cells.

Amnion and chorion cells have distinct and important roles in maintaining the integrity of the fetal membranes. The amnion layer provides the greatest tensile strength of the fetal membrane layers [46]. The chorion layer serves an important role in the maintenance of pregnancy by providing defense against infection and regulating apoptosis and inflammation [5, 47]. Our previous research demonstrated that infection accelerates apoptosis in the chorion layer in both term and PPROM subjects [48–50]. Taken together, these findings indicate that the preferential adherence of *U*. *parvum* to amnion and chorion cells provides a potential underlying mechanism of infection-induced fetal membrane rupture.

A standard bacterial inoculum is critically important for *in vivo* and *in vitro* studies. We have previously demonstrated that the quantity of bacteria present in the fetal membranes is correlated with chorion thinning suggesting that bacterial presence may incrementally evoke a host response that leads to chorion cell death and tissue degradation [5]. There is now evidence that the dose and variation of the multiple-banded antigen (MBA) of *U*. *parvum* might affect the severity of chorioamnionitis in pregnant sheep [51]. Therefore, we optimized the dose of *U*. *parvum* and treatment duration to avoid cell death in this current study. The optimized dose of 5x10^6^ CFU was chosen because: 1. the consistent and effective induction of IL-8, COX2, PGE2 and MMP9 in these cultures; 2. cell death was not observed in the treatment duration; 3. the MOI (~10: 5x10^6^ *U*. *parvum* to 5x10^5^ fetal membrane cells) was within the range of positive adherence to fetal membrane cells; and 4. the MOI of *U*. *parvum* in this study is consistent with other studies such as the study using human amniotic epithelial. In the study, 10^8^ CFU *U*. *parvum* was exposed to 10^7^ amniotic epithelial cells with a MOI of 10 [52]. To ensure a standard *U*. *parvum* inoculum, numerous aliquots of *U*. *parvum* concentrate from a single passage with a given CFU were frozen at -80°C, so that for each of the different experiments, equivalent quantities of *U*. *parvum* were utilized. We kept an aliquot of the *U*. *parvum* resuspension for quantification for each experiment. Consistent culturing conditions and quantification results ensured that the same *U*. *parvum* inoculum, 5x10^6^ CFU, used for each of the experiments for the amnion and chorion. Experimental procedure was standardized and the experiments can be directly compared.

Using this model, we demonstrated that exposure of *U*. *parvum* produced a robust pro-inflammatory state including IL-8, COX-2, PGE_2_ and MMP9 in primary human amnion and chorion cells. Up-regulation of IL-8, COX-2 and MMP9 can be associated with labor and play a critical role in fetal membrane infection and rupture. IL-8 concentration in amniotic fluid has been previously reported to positively correlate with *U*. *parvum* concentration [53], which is supported by our current and previous [18] findings *in vitro* along with another study [52]. Limited studies suggest several mechanisms through which *U*. *parvum* activates host inflammatory pathways. *U*. *parvum* can activate NF kappa β through the toll-like receptor 1, 2, and 6 [54]. The cell surface-associated MBA of *U*. *parvum* has been suggested to be one of several factors recognized by toll-like receptors 2/6 or 9 in amnion epithelial cells to induce inflammatory responses including IL-8 [52]. *Ureaplasmas* express phospholipases A and C, which could in turn alter prostaglandins synthesis-a known trigger of labor [41].

P4 is a clinically relevant therapeutic used to prevent PTB and PPROM. Mechanistically, it has been proposed to attenuate inflammatory responses in the cascade of events leading to adverse pregnancy outcomes. Previously, it had been shown that P4 elicits an inhibitory effect upon lipopolysaccharide (LPS)-induced innate immune response including tissue necrosis factor α (TNF-α) and IL-6 but not IL-8 in pre-labor human amniotic epithelium [55]. Another study found that progesterone represses IL-1β-induced IL-8 and COX-2 expression in human amnion epithelial cells [56]. We assessed the cell-specific effects of P4 on *U*. *parvum–*induced inflammation in amnion and chorion cells, predicting that P4 would attenuate inflammation induced by *U*. *parvum* in the amnion and chorion. Using concentration of P4 comparable to levels in amniotic fluid in the second half of pregnancy [57] and comparable with previous publications [55, 56], P4 did not significantly inhibit *U*. *parvum-*induced IL-8, COX-2 and PGE_2_ expression in amnion cells. P4 modestly attenuated IL-8 and COX-2 mRNA expression but not IL-8 and PGE_2_ protein concentration in chorion cells. The local concentration of P4 in fetal membranes may be higher than what has been measured in amniotic fluid [58, 59]. However non-specific steroid effects due to changes in cell membrane fluidity can occur with doses of progesterone in excess of micromolar concentrations [60], making assays using high concentrations difficult to interpret. Peltier *et al* observed that a high concentration of P4 (10 mg/mL) enhanced *U*. *urealyticum*-induced IL-8 production inflammation [61]. However, P4 at concentration more in line with clinically observed levels did not affect heat-killed *U*. *parvum* induced IL-8 production in Peltier’s study [61]. The combination of our data and those of Peltier suggest that P4 has minimal effects on the *Ureaplasma*-induced IL-8 inflammatory response at physiological concentrations in studied cells. Overall, our finding suggests that P4 might not play a critical role in modifying *U*. *parvum*-induced IL-8 or PGE_2_ production in fetal membranes.

In the intermediate to terminal events leading up to PPROM, COX-2 activation, PGE2 production, and metalloprotease activation are emerging as key events. A recent study suggested that PGE_2_ may mediate the selective increase in MMP9 activity after exposure of trophoblast cells to LPS [62]. IL-8 is also capable of increasing the production of the MMPs in fetal membranes [63]. Therefore, the excessive local production of COX-2 and consequent PGE_2_ production and IL-8 production following *U*. *parvum* exposure might contribute to MMP9 activation. MMP9 is clinically important in the pathophysiology of PPROM. Increased MMP9 activity leads to matrix protein degradation, resulting in cell detachment from the basement membrane, apoptotic cell death and a reduction in tissue tensile strength [23, 64, 65]. Inhibition of MMP9 activity by medroxyprogesterone acetate (MPA) has previously been described in first trimester cytotrophoblast cells [34]. In our cell-specific studies, P4 produced significant inhibition of MMP9 mRNA induced by *U*. *parvum* exposure in chorion cells but had no significant effect in amnion cells.

Together, these findings suggest that progesterone opposes various biochemical effects on inflammatory responses under different stimuli or in different cell types of the fetal membranes.

Although P4 effects are modest in *U*. *parvum* induced inflammation in fetal membrane cells, it is intriguing to note that loss of PGRMC1 expression is associated with significantly increased COX-2 and MMP9 mRNA expression following *U*. *parvum* exposure in chorion cells and increased MMP9 activity and decreased IL-8 protein secretion in amnion cells. The partial attenuation of MMP9 and COX2 mRNA expression in chorion cells by P4 appears to be PGRMC1 dependent. PGRMC1 is a single transmembrane bound receptor with an affinity for P4 of 11 nM [66]. Its signaling mechanism is unclear although motives for interaction with various kinases have been predicted from its amino acid sequence [67]. There is evidence that it activates JAK/STAT and PI3kinase pathways as well as regulating calcium influx [68]. Based on the different cellular location of PGRMC1 in amnion and chorion cells described in our results, it appears that PGRMC1 will likely have multiple sites of action within fetal membrane cells. It is therefore not surprising that PGRMC1 plays different roles in these cells. We previously demonstrated that PGRMC1 expression appears to be actively regulated in fetal membranes during pregnancy [31, 40] and mediates the action of progesterone, especially in amnion and chorion cells which lack nuclear progesterone receptors (PGRs) [69]. Additionally, PGRMC1 expression appears to be diminished in PPROM subjects [31]. Our current finding indicates that fetal membranes with diminished PGRMC1 might be more susceptible to *U*. *parvum* induced MMP9 and COX-2 production and MMP9 activity in fetal membranes. Together with its role in promoting cell survival [70], PGRMC1 could potentially be an attractive target for anti-infective prophylaxis and therapy for fetal membrane infection and PPROM.

In summary, we have shown *U*. *parvum* preferentially adheres to and induces inflammatory responses including IL-8, COX-2, PGE_2_ and MMP9 in chorion and amnion cells. P4 had differential effects on inflammation in amnion and chorion cells with partial attenuation of *U*. *parvum*-induced MMP9, IL-8 and COX2 mRNA expression in chorion cells, but demonstrated no effects on *U*. *parvum* induced inflammation in amnion cells. A significant role of PGRMC1 was identified in the inhibition of *U*. *parvum-*stimulated COX-2 and MMP9 mRNA expression in chorion cells and MMP9 activity in amnion cells. This data suggests that fetal membranes with diminished PGRMC1, such as in PPROM, might be more susceptible to *U*. *parvum*-induced COX-2, MMP9 production and MMP9 activity, and ultimately membrane weakening. Further molecular resolution of the interactions between *U*. *parvum* and the cells of the fetal membranes, *U*. *parvum* virulence factors, and the role of PGRMC1 in modifying the response to *U*. *parvum* will likely yield novel points to predict risk for PTB and PRROM and for therapeutic interventions to limit disease onset and progression.
